# Burnout and Mental Health Challenges Among Emergency Nurses in Sfax

**DOI:** 10.1192/j.eurpsy.2026.11683

**Published:** 2026-07-31

**Authors:** A. Kchaou, A. Triki, F. Dhouib, N. Kotti, M. Hajjaji, K. J. Hammami

**Affiliations:** Occupational Health, Hedi Chaker University Hospital, Sfax, Tunisia

## Abstract

**Introduction:**

Emergency departments represent high-demand environments where psychological strain often exceeds measurable physical workload. Healthcare staff in these settings are particularly vulnerable to stress, burnout, and mental health deterioration

**Objectives:**

This study aimed to assess the prevalence of poor mental health and burnout among emergency nurses in Sfax and to examine their association.

**Methods:**

We conducted a cross-sectional descriptive and analytical study in the emergency services (EMS) of two university hospitals in Sfax. Data were collected using a self-administered questionnaire that included socio-demographic and professional information, along with the General Health Questionnaire-12 (GHQ-12), and the Maslach Burnout Inventory.

Burnout (BO) was defined as an emotional exhaustion score ≥27, depersonalization ≥10, and professional accomplishment ≤45. Whereas, Poor mental health was defined by a GHQ-12 score ≥2.

**Results:**

A total of 40 nurses completed the questionnaire, with a sex ratio of 2.1. The average age was 34 ± 5 years. Hypothyroidism was the most common medical history (7.1%). Among the participants, 81% worked rotating shifts, averaging two night shifts per week.

Poor mental health was reported in 62% of nurses. Burnout prevalence was 58.2%, with high levels of emotional exhaustion (50.8%), depersonalization (81%), and reduced personal accomplishment (57.1%). A significant association was found between burnout and poor mental health (73.4% vs. 31.6% ; p = 0.001).

**Conclusions:**

Burnout and poor mental health are highly prevalent among emergency nurses in Sfax. The imbalance between effort and perceived reward has a significant impact on the mental health of emergency staff. Therefore, it is crucial to establish regular recognition systems for completed work and strengthen psychological support to help protect their mental well-being.

**Disclosure of Interest:**

None Declared

